# EXPLORING THE EFFECTS OF TRAUMA ON ADOLESCENT BRAIN AGING: A PRELIMINARY NEUROIMAGING STUDY

**DOI:** 10.51894/001c.123122

**Published:** 2024-08-31

**Authors:** Shravya Chanamolu

**Affiliations:** 1 College of Osteopathic Medicine Michigan State University

## 95

### INTRODUCTION/BACKGROUND

Exposure to traumatic events during childhood, such as violence, influences brain and behavioral development, which may increase risk of neuropsychiatric disorders. Research shows that childhood trauma may alter the pace of structural neurodevelopment across adolescence, a period of dynamic changes in neural circuitry. This preliminary study explores the impact of childhood trauma exposure on brainAGE — a neuroimaging biomarker of brain aging — and its deviation from chronological age.

### OBJECTIVES/HYPOTHESIS

The primary objective of this project is to identify associations between trauma exposure during childhood/adolescence and the difference between brainAGE and chronological age.

### METHODS

We report preliminary data from an ongoing neuroimaging study of 72 adolescents recruited from an urban area with high rates of trauma exposure (n = 72; 56.90% male, age M±SD = 13.11±2.31 years). Adolescents provided self-reports on number of potentially traumatic events experienced. T1-weighted magnetic resonance images (MRI) data was also acquired and brainAGE was calculated using a machine learning algorithm. We explored associations between number of traumas and brainAGE, and the difference between brainAGE and chronological age.

### RESULTS

The youth in this sample reported M±SD = 4.04±3.02 traumatic events, with 80% of them reporting at least one traumatic event. The brainAGE was significantly higher than chronological in this adolescent sample by M±SD = 6.45±2.39 years (p<0.001). We did not observe a significant association between the number of potentially traumatic events and the accelerated brainAGE (p>0.05).

### DISCUSSION/CONCLUSIONS

We found that there is a significant acceleration in brainAGE compared to chronological age, but there was no evidence of the number of childhood traumas influencing brainAGE in this preliminary study. However, future analyses in larger studies will shed light on which aspects of trauma exposure early in life may accelerate, delay, or cause a divergence in structural neurodevelopment.

